# Discovery of pharmacological effects and targets of *Citri Grandis Exocarpium based* on SYSTCM and virtual screening

**DOI:** 10.29219/fnr.v68.10618

**Published:** 2024-06-20

**Authors:** Qinqi Feng, Xinyang Shu, Hanyu Fang, Xiaoxi Shi, Yanling Zhang, Hongchun Zhang

**Affiliations:** 1 Beijing University of Chinese Medicine, Beijing, China; 2 Department of Traditional Chinese Medicine for Pulmonar y Diseases, National Center for Respirator y Medicine, National Clinical Research Center for Respirator y Diseases, Institute of Respirator y Medicine, Center of Respirator y Medicine, China-Japan Friendship Hospital Chinese Academy of Medical Sciences, Beijing, China; 3 Key Laboratory of TCM-information Engineer of State Administration of TCM, School of Chinese Materia Medica, Beijing University of Chinese Medicine, Beijing 102488, China

**Keywords:** Citri Grandis Exocarpium, *Antiallergic effect*, *TCM*, *SYSTCM*

## Abstract

*Citri Grandis Exocarpium* (Huajuhong, CGE) is the peel of the unripe fruits of *Citrus grandis* ‘*Tomentosa’ and Citrus grandis* (L.) Osbeck, which is commonly used in the clinic for the treatment of cough and indigestion. The pharmacological mechanism of CGE is unclear. In this study, the pharmacological effect of CGE was predicted by System Traditional Chinese Medicine (SYSTCM), which integrated the pharmacological effect prediction approach by artificial intelligence into the systemic traditional Chinese medicine (TCM) platform. The main pharmacological effect of CGE was antiallergy, promoting bile, blood lipid regulation, cardiotonics, diuresis, and antiarrhythmia by prediction of SYSTCM. *In vitro* cell experiments were carried out to identify the antiallergic effect of CGE. Extracts of *Citri Grandis Exocarpium* (ECGE) inhibited lipopolysaccharide-induced cell injury and nitric oxide release in RAW264.7 cells. ECGE and naringin-inhibited immunoglobulin E-induced cell degranulation in RBL-2H3 cells. Target profiling, protein interaction network, and molecular docking of compounds from CGE indicated that mitogen-activated protein kinase 14 (MAPK14) and matrix metalloprotease 9 (MMP9) were key potential targets of CGE with antiallergic activity. This study identified and validated the antiallergic effect of CGE by combining SYSTCM, cell experiments, and virtual screening, which provided a new paradigm and approach for studying the pharmacological effect and mechanism of TCM.

## Popular scientific summary

- Anti-allergy, promoting bile, blood lipid regulation, cardiotonics, diuresis and anti-arrhythmia are the main pharmacological effects of CGE by prediction of SYSTCM.
- Naringin, chryso-obtusin glucoside, and rubrofusarin-6-O-β-D-gentiobioside were the main active compounds of CGE that interacted with MAPK14 and MMP9.
- MAPK14 and MMP9 were potential key targets for the anti-allergic effects of CGE complexes through target analysis, protein interaction networks and molecular docking.

*Citri Grandis Exocarpium* (Huajuhong, CGE) is derived from *Citrus grandis ‘Tomentosa’ and Citrus grandis* (L.) *Osbeck* (1). CGE is an essential TCM used to treat diseases of the respiratory and digestive systems (2, 3). Naringin is the index component of CGE for quality control in the Chinese Pharmacopoeia (Edition 2020). The content of naringin can reflect the quality of different grades of CGE decoction pieces (4). CGE has a wide range of pharmacological effects, including hypolipidemic, antimicrobial, anti-inflammatory, anticancer, and so on (5). However, the main pharmacological effects of CGE are not confirmed at present, and the mechanism of CGE for multiple pharmacological effects is not clear.

In this study, System Traditional Chinese Medicine (SYSTCM) (http://systcm.cn) with the prediction approach of pharmacological effect by artificial intelligence was utilized to predict the pharmacological effect of CGE. The main pharmacological effects of CGE, antiallergic, and anti-inflammatory effects were validated in lipopolysaccharide (LPS)-induced RAW264.7 and immunoglobulin E (IgE)-induced RBL-2H3 cells. The phenotype related to antiallergic effect was detected with the treatment of extracts of *Citri Grandis Exocarpium* (ECGE). The targets interacted with compounds from CGE were predicted based on pharmacophore-based virtual screening and analysis of protein interaction network (PIN) and validated by molecular docking. The pharmacological effect and mechanism of CGE were revealed by combining SYSTCM and virtual screening.

## Materials and methods

### Materials

RBL-2H3 cell was purchased from Shanghai Ze Ye Biotechnology Co. Ltd, and RAW264.7 cell was purchased from the American Type Culture Collection. Dulbecco’s Modified Eagle’s Medium (DMEM) was purchased from Corning Incorporated, and fetal bovine serum and penicillin-streptomycin were purchased from Gibco. Naringin was purchased from Shanghai Yuanye Bio-Technology Co., Ltd. (Shanghai, China) with purities higher than 98%. CGE was purchased from Hebei Renxin Pharmaceutical Co., Ltd. 3-(4,5)-dimethylthiahiazo (-z-y1)-3,5-di- phenytetrazoliumromide (MTT) and LPS were purchased from Sigma-Aldrich. NO kit was purchased from Applygen Technologies Inc. Anti-dinitrophenyl-IgE and DNP-BSA were purchased from Sigma-Aldrich. PNAG was purchased from J&K Scientifc.

### Pharmacological prediction of CGE based on SYSTCM

A total of 79 compounds from CGE were collected from the databases of TCMSP (6), TCMD, ETCM (7), and HERB (8). Compounds were input into the identification model of pharmacological effects using a convolutional neural network (9) in SYSTCM. The 40 pharmacological effects of compounds from CGE were predicted, and the nature of the 40 pharmacological effects was ordered based on the number of compounds with potential pharmacological effects.

### Cell culture

RBL-2H3 and RAW264.7 were cultured in DMEM with 10% fetal bovine serum and 1% penicillin-streptomycin. All cells were cultured at 37°C and 5% CO_2_ in a humidified atmosphere.

### Preparation of ECGE

CGE (100 g) was extracted and refluxed with 1 L water for 3 h and concentrated for 200 mL (0.5 g/mL). ECGE was further freeze dried and dissolved in PBS at a concentration of 100 mg/mL.

### Cell viability assay

MTT assay was utilized to identify the appropriate dose of different treatments for *in vitro* assay. About RBL-2H3 (1.5 × 10^4^ cells/well) and RAW264.7 (6 × 10^4^ cells/well) are seeded and cultured in 96-well plates overnight. Cells are treated with five doses of different treatments for further 24 h. Then, the medium was removed, and MTT (0.5 mg/mL) was added and cultured with cells for 4 h. DMSO was utilized to terminate reaction, and the absorbance at 490 nm was measured using FlexStation 3 (Molecular Devices, San Jose, CA). The cell survival rates are calculated as [(OD_drug_ − OD_blank_)/ (OD_control_ − OD_blank_)] × 100%.

### Nitric oxide (NO) production

RAW264.7 (6 × 10^4^ cells/well) were seeded and cultured into 96-well plates overnight, and the cell medium was replaced with medium supplemented with ECGE with or without 0.1 μg/mL LPS for 24 h. Griess approach was utilized to detect the concentration of NO release. The supernatant (50 μL) was incubated with Griess reagent for 5 min, and the plate was read on FlexStation 3 at 540 nm. The concentration of NO production for each sample was detected using a standard curve of NaNO_2_.

### IgE-induced degranulation assay

RBL-2H3 (1.5 × 10^4^ cells/well) were seeded and cultured into 96-well plates overnight, and the cell medium was replaced with medium supplemented with or without 400 ng/mL anti-dinitrophenyl-IgE (anti-DNP-IgE) for sensitization. After sensitizing for 24 h, the cells were pretreated with ECGE or naringin for 1 h at 37°C. After incubation, the cells were stimulated with or without 400 ng/mL DNP-bovine serum albumin (DNP-BSA) for 30 min at 37°C and cooled to 0°C in an external ice bath for 10 min.

The supernatant (50 μL) was incubated for 1.5 h at 37°C with 50 μL of p-nitrophenyl-N-acetyl-β-D-glucosaminide (PNAG, 4 M). Afterward, the reaction was terminated by the addition of 200 μL of 0.1 M sodium carbonate buffer. The generation of p-nitrophenol in supernatant was utilized to quantify the activity of β-hexosaminidase (β-HEX) by absorbance at 405 nm. The released rate of β-HEX (%) was calculated as (ODdrug − ODcontrol)/(ODmodel − ODcontrol) × 100%.

### Target identification of compounds from CGE based on pharmacophore

The 3D structure of 79 compounds from CGE was generated and fully minimized in CHARMm force field with MMFF94 partial charge. Reverse target identification of compounds from CGE was performed based on the ligand profiler module in Discovery studio 4.0 (Accerlrys Inc. San Diego, CA). Diverse conformations of compounds were generated by BEST mode with 255 conformations, and the relative energy threshold was less than 20.0 kcal/mol. The targets of compounds were predicted by the pharmacophore database of pharmaDB containing 7028 pharmacophore models with the flexible searching method. Fit value was utilized to evaluate the overlap degree of compounds and pharmacophore. The relationships of compounds and targets with fit value more than 0.9 were considered as the active compounds and potential targets.

### PIN of CGE

The protein–protein interaction (PPI) information of potential targets was derived from String 12.0 (http://string-db.org). PPIs of potential targets with interaction score more than 0.4 were extracted to construct PIN by Cytoscape 3.10.0. The largest connected subgraph was obtained as the PIN of CGE, and the topological parameters of PIN were calculated for the identification of potential targets of CGE.

### Molecular docking

According to the reverse target identification, the relationships of active compounds and potential targets were further identified by molecular docking. The crystal structures of potential targets were downloaded from Protein Data Bank (PDB, http://www.rcsb.org). The common problems were automatically solved, including cleaning crystallographic waters and adding hydrogens. The binding sites of proteins were defined from the ligands complexed in crystal structures (initial ligands). Molecular docking was performed by CDOCKER algorithms, the initial ligands were extracted from the binding sites, and re-docked into the sites for calculating the root mean square deviation (RMSD). RMSD less than 2.00Å indicated docking algorithm was suitable and could reappear the binding of targets and ligands. Active compounds from CGE were docked into binding sites to calculate -cDOCKER interaction energy and analyze binding poses and key residues.

## Results

### Prediction of pharmacological effects of CGE

**Fig. 1:**
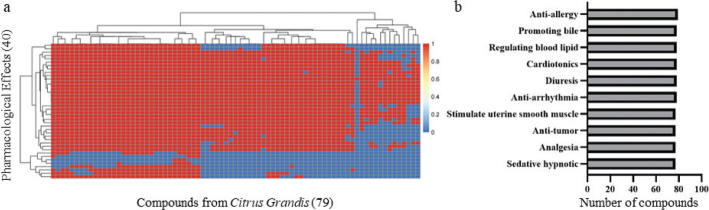
Prediction of pharmacological effects of CGE based on SYSTCM. (a) The prediction heatmap of pharmacological effects of CGE. The color of each spot in the heatmap corresponds to the prediction results (1 or 0) of each pharmacological effect for compounds. (b) Top 10 prediction results of pharmacological effects of CGE based on the number of hit compounds.

Total 79 compounds from CGE were collected from TCM databases, and 40 pharmacological effects of 79 compounds from CGE were predicted by the convolutional neural network in SYSTCM (Fig. 1a and Table S1). Among the potential pharmacological effects of CGE, the top 6 were antiallergy, promoting bile, regulating blood lipid, cardiotonics, diuresis, and anti-arrhythmia, which is consistent with clinical usage (Fig. 1b). The clinical usage of CGE for the treatment of cough is highly related to the pharmacological effect of anti-allergy (ranking 1/40), which was also coexisted with anti-inflammatory effects (ranking 15/40). Therefore, the pharmacological effect of anti-inflammatory and antiallergy effects of CGE was further validated by *in vitro* experiments.

### Anti-inflammatory effects of CGE

**Fig. 2:**
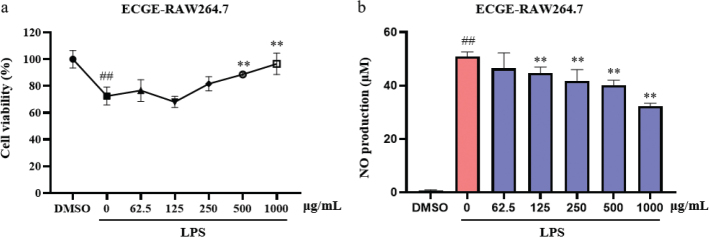
Anti-inflammatory effects of ECGE in RAW264.7 cells. Cell viability (a) and NO production (b) of LPS-induced RAW264.7 cells treated with different concentrations of ECGE (62.5, 125, 250, 500, and 1000 μg/mL) for 24 h (*n* = 3).

Anti-inflammatory effects of CGE were validated in LPS-induced macrophages of RAW264.7 cells, which were classic inflammatory cell models. RAW264.7 cells were simulated by LPS for 24 h to induce cell injury (Fig. 2a). There was no significant decrease in cell viability treated with ECGE (62.5–250 μg/mL). Cell injury of RAW264.7 was significantly protected with the treatment of ECGE (500–1000 μg/mL). Nitric oxide is a key biomarker of inflammation, and the reduction of NO can be used to characterize the improvement of inflammation. RAW264.7 cells were simulated by LPS for 24 h to induce NO production (Fig. 2b). The NO production was significantly reduced by the treatment of ECGE (125–1000 μg/mL), which indicated ECGE had the anti-inflammatory effects.

### Antiallergic effects of CGE and naringin

**Fig. 3:**
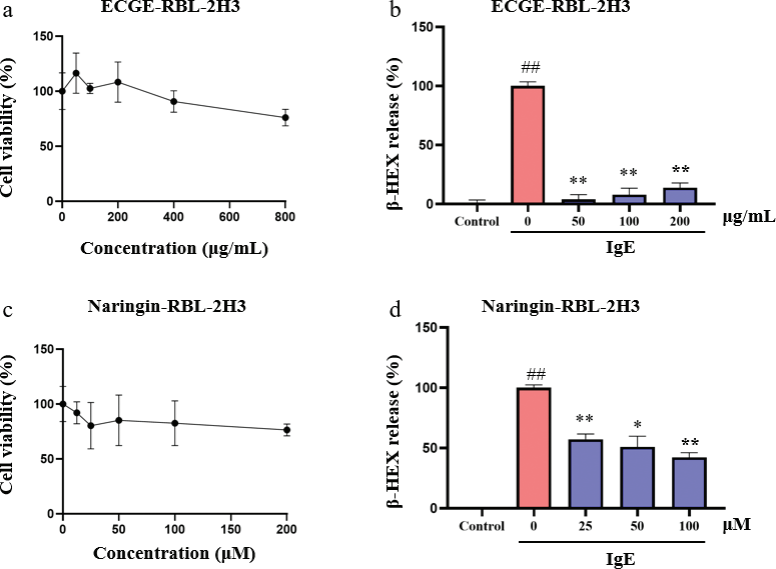
Anti-allergic effects of ECGE and naringin in RBL-2H3 cells. Cell viability (a) and β-HEX release (b) of IgE-induced RBL-2H3 cells treated with different concentrations of ECGE (*n* = 3). Cell viability (c) and β-HEX release (d) of IgE-induced RBL-2H3 cells treated with different concentrations of naringin (*n* = 3).

Antiallergic effects of CGE and naringin were validated in IgE-induced RBL-2H3 cells, which were classic allergic cell model with phenotype of degranulation. RBL-2H3 cells were treated with ECGE for 24 h and had little cytotoxicity within the analyzed concentration range (50–800 μg/mL, Fig. 3a). RBL-2H3 cells were simulated by anti-DNP-IgE for 24 h and following by DNP-BSA for 30 min to induce cell degranulation (Fig. 3b). Cell degranulation of RBL-2H3 was significantly inhibited with the treatment of ECGE (50–200 μg/mL). RBL-2H3 cells were treated with naringin for 24 h and had little cytotoxicity within the analyzed concentration range (12.5–200 μM, Fig. 3c). RBL-2H3 cells were simulated by IgE to induce β-HEX release (Fig. 3d). β-HEX release was significantly reduced by the treatment of naringin (25–100 μg/mL), which indicated naringin had the anti-allergic effects.

### Target profiles, PIN, and molecular docking of compounds from CGE

**Fig. 4:**
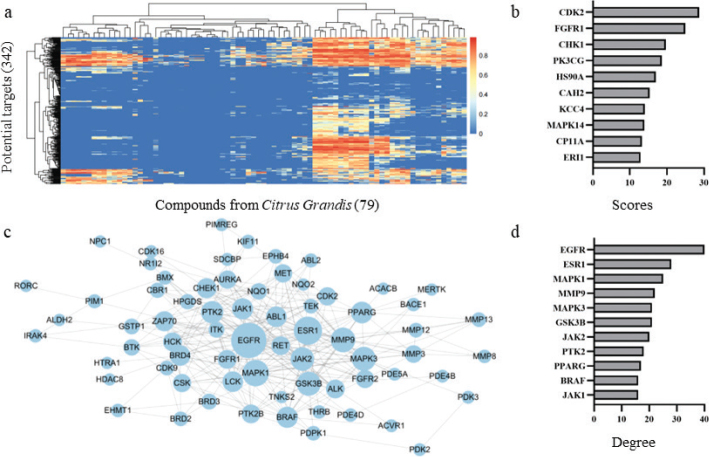
Target profiles and network pharmacology of compounds from CGE. (a) The heatmap of target profiles of compounds from CGE. The color of each spot in the heatmap corresponds to the fit value of potential targets for active compounds. (b) Top 10 potential targets of CGE based on sum of fit value of active compounds. (c) Protein interaction network (PIN) of potential targets of CGE. (d) Top 10 potential targets of CGE based on degree in PIN.

Reverse target profiles based on pharmacophore models were performed to reveal the anti-inflammatory and antiallergy mechanism of CGE and discover the potential targets of compounds from CGE. A total 342 targets were hit by 79 compounds from CGE (Fig. 4a and Table S2). According to filter and sum of fit value, 119 targets were identified as the potential targets of CGE with fit value more than 0.9 of one compound (Table S3). Top 10 potential targets of CGE with the maximum sum of fit values were CDK2, FGFR1, CHK1, PK3CG, HS90A, CAH2, KCC4, MAPK14, CP11A, and ERI1 (Fig. 4b).

PIN of CGE with 66 nodes and 264 interactions was constructed to reveal the mechanism of CGE (Fig. 4c). Topological parameter of PIN was analyzed, and the degree of each node was calculated to analyze the potential targets. Top 10 potential targets of CGE with the maximum degree were EGFR, ESR1, MAPK1, MMP9, MAPK3, GSK3B, JAK2, PTK2, PPARG, BRAF, and JAK1 (Fig. 4d). Total 20 potential targets analyzed by two approaches were searched in PubMed database using keywords related to anti-inflammatory and antiallergic effects (Table S4), and key targets of MAPK14 (10) and MMP9 (11) regulated by CGE with anti-inflammatory and antiallergic effects were identified and validated by molecular docking.

**Fig. 5:**
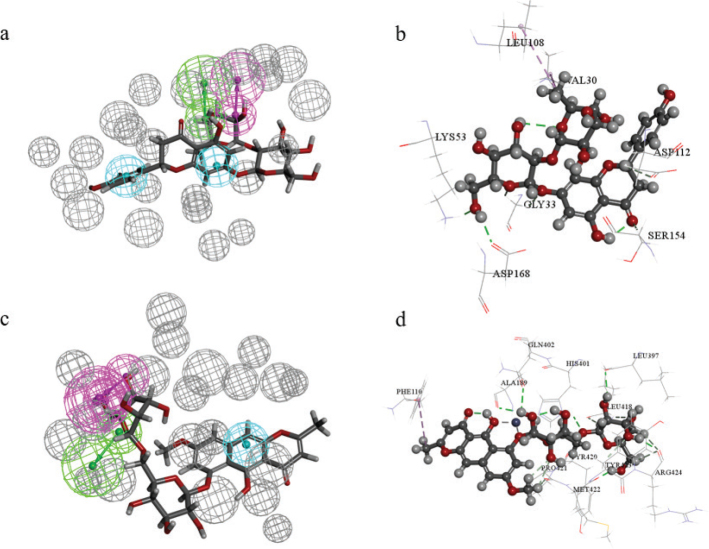
Interaction analysis of potential targets and compounds from CGE. (a) Mapping graph of naringin and MAPK14 pharmacophore. (b) Docking result between naringin and MAPK14. (c) Mapping graph of rubrofusarin-6-O-β-D-gentiobioside and MMP9 pharmacophore. (b) Docking result between rubrofusarin-6-O-β-D-gentiobioside and MMP9.

Molecular docking was performed to refine the interaction between 79 compounds from CGE and MAPK14 or MMP9. Binding sites of MAPK14 and MMP9 were identified based on initial ligands with RMSD less than 2.00Å during CDOCKER process (Table S5). The compounds with -CDOCKER INTERACTION ENERGY higher than initial ligands were regarded as the potential active compounds (12). Combining the scores of pharmacophore and molecular docking, potential active compounds were identified for MAPK14 and MMP9 (Table S6). Naringin, chryso-obtusin glucoside, and rubrofusarin-6-O-β-D-gentiobioside were potential active compounds interacted with MAPK14 with -CDOCKER INTERACTION ENERGY higher than initial ligand in docking and fit value higher than 0.9 in pharmacophore. Naringin interacted with MAPK14 by hydrogen bond interaction with GLY33, LYS53, ASP112, SER154, ASP168, and hydrophobic interactions with VAL30 and LEU108 (Fig. 5a and b). The -CDOCKER INTERACTION ENERGY of naringin, chryso-obtusin glucoside, and rubrofusarin-6-O-β-D-gentiobioside were 54.91, 61.65, and 61.38 kcal/mol for MAPK14. Rubrofusarin-6-O-β-D-gentiobioside was potential active compound interacted with MMP9 with -CDOCKER INTERACTION ENERGY higher than initial ligand in docking and fit value higher than 0.9 in pharmacophore. Rubrofusarin-6-O-β-D-gentiobioside interacted with MMP9 by hydrogen bond interaction with ALA189, LEU397, HIS401, GLN402, LEU418, TYR420, PRO421, MET422, TYR423, ARG424, and hydrophobic interactions with PHE110 (Fig. 5c and d). The -CDOCKER INTERACTION ENERGY of rubrofusarin-6-O-β-D-gentiobioside was 79.48 kcal/mol for MMP9. Naringin, chryso-obtusin glucoside, and rubrofusarin-6-O-β-D-gentiobioside were potential active compounds from CGE with the anti-inflammatory and antiallergic effects through MAPK14 and MMP9.

## Discussion

In this study, we utilized artificial intelligence models in SYSTCM to predict the antiallergic effects of Citrus grandis extract (CGE) and validated these predictions through in vitro experiments. We observed that MAPK14 and MMP9 are key potential targets of CGE with antiallergic activity.

Studies of the antiallergic effects and mechanism of CGE were insufficient, but multiple studies reported that naringin had antiallergic effects. Naringin inhibited airway resistance and eosinophil count in OVA-induced mice and regulated multiple allergic cytokines, including interleukin-4, INF-gamma, and MMP9 (13, 14). Meanwhile, naringin promotes the proliferation of airway epithelial cells via activation of taste receptor type 2 member (TAS2R) signaling pathways (15). In this study, we identified the antiallergic effects of CGE and naringin by IgE-induced degranulation assay. Virtual screening and PIN indicated that naringin, chryso-obtusin glucoside, and rubrofusarin-6-O-β-D-gentiobioside were the main active compounds from CGE interacted with MAPK14 and MMP9, which provides the key mechanism of CGE for the treatment of respiratory diseases.

Allergic diseases are immune imbalances caused by a combination of genes and the environment. The process of the organism becoming allergic involves the activation of genes, a variety of cytokines, and enzymes (16). It is well known that MAPK14 (also known as protein kinase p38α) is a member of the mitogen-activated protein kinase family that is activated by environmental and endogenous physiological stimuli associated with tissue injury and infection (17). The p38 mitogen-activated protein kinase (MAPK) family plays an important role in the inflammatory response in a wide range of disorders (18). The MAPK pathway promotes inflammatory responses in various cells such as lymphocytes in the lung and bronchi during the process of allergic asthma (19). In allergen-exposed skin, MAPK14 regulates epidermal inflammatory genes by phosphorylating p63 leading to allergic skin inflammation (17). Th2 regulates antiparasitic and allergic responses, and MAPK14 modulates Th2 responses in vitro and in vivo by regulating a variety of TCR-related signals (10). MMP9 is closely associated with a variety of allergic diseases (20), including allergic nasal polyps (21), asthma (22), allergic bronchopulmonary aspergillosis (23), atopic dermatitis (24), pollen allergy (25), and others. Mast cells are closely related to MMP9 (26), which is involved in mast cell activation (27). Zhang et al. identified MMP9 as a new biomarker of early efficacy of sublingual immunotherapy for allergic rhinitis by serum proteomics (28). Notably, MMP9 is equally associated with Th2-mediated acute inflammation in asthma (29). MMP9 and MAPK14 might also be related due to MAPK14 and MMP-9 being associated with cancer development (30). While studies have demonstrated that some herbal medicines and their components can inhibit inflammatory cytokines, MMP9 and MAPK14 are one of their therapeutic targets (31, 32).

TCM has advantages for the treatment of complex diseases, including respiratory disease (33, 34), cardiovascular disease (35, 36), and so on. However, complex pharmacological effects and unclear mechanisms reminder the development of TCM. The mechanism of TCM is essential for the dissemination and utilization of TCM all over the world. Artificial intelligence technology provided a new method to discover and identify the pharmacological effect of TCMs. In this study, artificial intelligence models in SYSTCM were utilized to predict the pharmacological effect of CGE and may provide a new perspective and application for the research of TCM.

CGE is the traditional homologue of food and medicine used in the protection and treatment of diseases of respiratory system. Pharmacological research indicated CGE has multiple pharmacological effects in multiple types of cells and signaling pathways of respiratory system. CGE and flavonoids had anti-inflammatory effects in xylene-induced mice models (37) and LPS-induced RAW264.7 and regulated the activation of MAPK and NF-κB signaling pathways (38). Aqueous extract of CGE also alleviated bleomycin-induced idiopathic pulmonary fibrosis in rat model, which related to the reduction of IL-6, IL-8, TGF-β1, and so on (4). In this study, we re-proved the anti-inflammatory effect in LPS-induced RAW264.7 and reveal the mechanism of CEG through active compounds and potential targets of MAPK14 and MMP9.

Actually, other new pharmacological effects of CGE predicted by SYSTCM should be further validated by pharmacological research. The potential targets predicted in this study should be further validated by in vitro and in vivo experiments. And the key compounds predicted by virtual screening should be further analyzed by HPLC for identification of the content of CGE.

In conclusion, six main pharmacological effects of CGE were predicted by artificial intelligence approach in SYSTCM. Wherein, antiallergy and anti-inflammatory effects of CGE were validated in LPS-induced RAW264.7 cells and IgE-induced RBL-2H3 cells. MAPK14 and MMP9 were identified as the main potential targets of naringin, chryso-obtusin glucoside, and rubrofusarin-6-O-β-D-gentiobioside from CGE by target profiles, PIN, and molecular docking. This study integrated SYSTCM, cell experiments, and virtual screening to discover and validate the pharmacological effects of TCM and provided a new paradigm for revealing the mechanism of TCM.

## Supplementary Material



## References

[1] Su C, Wong K-L, But PP-H, Su W-W, Shaw P-C. Molecular authentication of the Chinese herb Huajuhong and related medicinal material by DNA sequencing and ISSR markers. J Food Drug Anal 2010; 18: 161–70. doi: 10.38212/2224-6614.2267

[2] Xu Z, Li J, Zhou K, Wang K, Hu H, Hu Y, et al. Exocarpium Citri Grandis ameliorates LPS-induced acute lung injury by suppressing inflammation, NLRP3 inflammasome, and ferroptosis. J Ethnopharmacol 2024; 329: 118162. doi: 10.1016/j.jep.2024.118162 38588989

[3] Deng G, Liu C, Zhao J, Wang M, Li Y, Yang M, et al. Exocarpium Citri Grandis alleviates the aggravation of NAFLD by mitigating lipid accumulation and iron metabolism disorders. J Ethnopharmacol 2023; 313: 116559. doi: 10.1016/j.jep.2023.116559 37116730

[4] Liu G, Li S, Zhang N, Wei N, Wang M, Liu J, et al. Sequential grade evaluation method exploration of Exocarpium Citri Grandis (Huajuhong) decoction pieces based on ‘network prediction → grading quantization → efficacy validation’. J Ethnopharmacol 2022; 291: 115149. doi: 10.1016/j.jep.2022.115149 35231589

[5] Tocmo R, Pena-Fronteras J, Calumba KF, Mendoza M, Johnson JJ. Valorization of pomelo (Citrus grandis Osbeck) peel: a review of current utilization, phytochemistry, bioactivities, and mechanisms of action. Compr Rev Food Sci Food Saf 2020; 19: 1969–2012. doi: 10.1111/1541-4337.12561 33337092

[6] Ru J, Li P, Wang J, Zhou W, Li B, Huang C, et al. TCMSP: a database of systems pharmacology for drug discovery from herbal medicines. J Cheminform 2014; 6: 13. doi: 10.1186/1758-2946-6-13 24735618 PMC4001360

[7] Xu H, Zhang Y, Liu Z, Chen T, Lv C, Tang S, et al. ETCM: an encyclopaedia of traditional Chinese medicine. Nucleic Acids Res 2019; 47: D976–82. doi: 10.1093/nar/gky987 30365030 PMC6323948

[8] Fang S, Dong L, Liu L, Guo J, Zhao L, Zhang J, et al. HERB: a high-throughput experiment-and reference-guided database of traditional Chinese medicine. Nucleic Acids Res 2021; 49: D1197–206. doi: 10.1093/nar/gkaa1063 33264402 PMC7779036

[9] Huo M, Peng S, Li J, Zhang Y, Qiao Y. A discovery strategy for active compounds of Chinese medicine based on the prediction model of compound-disease relationship. J Oncol 2022; 2022: 8704784. doi: 10.1155/2022/8704784 35847368 PMC9286898

[10] Xia T, Ma J, Sun Y, Sun Y. Androgen receptor suppresses inflammatory response of airway epithelial cells in allergic asthma through MAPK1 and MAPK14. Hum Exp Toxicol 2022; 41: 9603271221121320. doi: 10.1177/09603271221121320 35982617

[11] Corry DB, Kiss A, Song LZ, Song L, Xu J, Lee SH, et al. Overlapping and independent contributions of MMP2 and MMP9 to lung allergic inflammatory cell egression through decreased CC chemokines. Faseb J 2004; 18: 995–7. doi: 10.1096/fj.03-1412fje 15059974 PMC2771179

[12] Qiao L, He Y, Huo X, Jiang L, Chen Y, Chen X, et al. Construction and evaluation of merged pharmacophore based on peroxisome proliferator receptor-alpha agonists. Chin J Chem Phys 2016; 29: 508–16. doi: 10.1063/1674-0068/29/cjcp1602025

[13] Xiong G, Liu S, Gao J, Shumin W. Naringin protects ovalbumin-induced airway inflammation in a mouse model of asthma. Inflammation 2016; 39: 891–9. doi: 10.1007/s10753-016-0321-7 26920847

[14] Lee C-M, Chang J-H, Jung I-D, Jeong Y-I, Tae N-K, Park H-j, et al. Naringin protects ovalbumin-induced asthma through the down-regulation of MMP-9 activity and GATA-3 gene. J Life Sci 2009; 19: 735–43. doi: 10.5352/JLS.2009.19.6.735

[15] Ni K, Guo J, Bu B, Pan Y, Li J, Liu L, et al. Naringin as a plant-derived bitter tastant promotes proliferation of cultured human airway epithelial cells via activation of TAS2R signaling. Phytomedicine 2021; 84: 153491. doi: 10.1016/j.phymed.2021.153491 33601237

[16] Wang J, Zhou Y, Zhang H, Hu L, Liu J, Wang L, et al. Pathogenesis of allergic diseases and implications for therapeutic interventions. Signal Transduct Targeted Ther 2023; 8: 138. doi: 10.1038/s41392-023-01344-4 PMC10039055 36964157

[17] Jiménez-Andrade Y, Hillette KR, Yoshida T, Kashiwagi M, Choo M-K, Liang Y, et al. The developmental transcription factor p63 is redeployed to drive allergic skin inflammation through phosphorylation by p38α. J Immunol 2022; 208: 2613–21. doi: 10.4049/jimmunol.2101160 35623662 PMC9308733

[18] Yong H-Y, Koh M-S, Moon A. The p38 MAPK inhibitors for the treatment of inflammatory diseases and cancer. Expert Opin Investig Drugs 2009; 18: 1893–905. doi: 10.1517/13543780903321490 19852565

[19] Bouazza B, Debba-Pavard M, Amrani Y, Isaacs L, O’Connell D, Ahamed S, et al. Basal p38 mitogen-activated protein kinase regulates unliganded glucocorticoid receptor function in airway smooth muscle cells. Am J Respir Cell Mol Biol 2014; 50: 301–15. doi: 10.1165/rcmb.2012-0522OC 24024586 PMC3930943

[20] Suzukawa M, Komiya A, Iikura M, Nagase H, Yoshimura-Uchiyama C, Yamada H, et al. Trans-basement membrane migration of human basophils: role of matrix metalloproteinase-9. Int Immunol 2006; 18: 1575–83. doi: 10.1093/intimm/dxl090 16985079

[21] Bugdayci G, Kaymakci M, Bukan N. Matrix metalloproteinase-9 (MMP-9) in allergic nasal polyps. Acta Histochem 2010; 112: 92–5. doi: 10.1016/j.acthis.2008.07.002 18835014

[22] Wenzel SE, Balzar S, Cundall M, Chu HW. Subepithelial basement membrane immunoreactivity for matrix metalloproteinase 9: association with asthma severity, neutrophilic inflammation, and wound repair. J Allergy Clin Immunol 2003; 111: 1345–52. doi: 10.1067/mai.2003.1464 12789238

[23] Gibson PG, Wark PAB, Simpson JL, Meldrum C, Meldrum S, Saltos N, et al. Induced sputum IL-8 gene expression, neutrophil influx and MMP-9 in allergic bronchopulmonary aspergillosis. Eur Respir J 2003; 21: 582–8. doi: 10.1183/09031936.03.00001803 12762339

[24] Devillers ACA, van Toorenenbergen AW, Klein Heerenbrink GJ, Muldert PGH, Oranje AP. Elevated levels of plasma matrix metalloproteinase-9 in patients with atopic dermatitis: a pilot study. Clin Exp Dermatol 2007; 32: 311–3. doi: 10.1111/j.1365-2230.2007.02378.x 17335547

[25] Inoue H, Mashimo Y, Funamizu M, Yonekura S, Horiguchi S, Shimojo N, et al. Association of the MMP9 gene with childhood cedar pollen sensitization and pollinosis. J Hum Genet 2012; 57: 176–83. doi: 10.1038/jhg.2011.148 22237587

[26] Kanbe N, Tanaka A, Kanbe M, Itakura A, Kurosawa M, Matsuda H. Human mast cells produce matrix metalloproteinase 9. Eur J Immunol 1999; 29: 2645–9. doi: 10.1002/(SICI)1521-4141(199908)29:08<2645::AID-IMMU2645>3.0.CO;2-1 10458779

[27] Xu L, Cai Z, Yang F, Chen M. Activation-induced upregulation of MMP9 in mast cells is a positive feedback mediator for mast cell activation. Mol Med Rep 2017; 15: 1759–64. doi: 10.3892/mmr.2017.6215 28259919

[28] Zhang Y, Shui J, Wang L, Wang F. Serum proteomics identifies S100A11 and MMP9 as novel biomarkers for predicting the early efficacy of sublingual immunotherapy in allergic rhinitis. Int Immunopharmacol 2023; 124: 110857. doi: 10.1016/j.intimp.2023.110857 37647677

[29] Hendrix AY, Kheradmand F. The Role of Matrix Metalloproteinases in Development, Repair, and Destruction of the Lungs. Prog Mol Biol Transl Sci 2017; 148: 1–29. 28662821 10.1016/bs.pmbts.2017.04.004

[30] Zeng W, Song Y, Wang R, He R, Wang T. Neutrophil elastase: from mechanisms to therapeutic potential. J Pharm Anal 2023; 13: 355–66. doi: 10.1016/j.jpha.2022.12.003 37181292 PMC10173178

[31] Li L, Jia Q, Zhang H, Yi L, Tang Y, Hu P, et al. Tissue lipidomics, network pharmacology, and molecular docking to explore the therapeutic mechanism of anthocyanins from Lycium ruthenicum Murr. against gouty arthritis. Food Funct 2023; 14: 7011–23. doi: 10.1039/D1FO04377C 37439115

[32] Huang X-J, Wang J, Muhammad A, Tong H-Y, Wang D-G, Li J, et al. Systems pharmacology-based dissection of mechanisms of Tibetan medicinal compound Ruteng as an effective treatment for collagen-induced arthritis rats. Journal of Ethnopharmacology 2021; 272: 113953. doi: 10.1016/j.jep.2021.113953 33610711

[33] Qiao L, Huang W, Zhang X, Guo H, Wang D, Feng Q, et al. Evaluation of the immunomodulatory effects of anti-COVID-19 TCM formulae by multiple virus-related pathways. Signal Transduct Target Ther 2021; 6: 577–9. doi: 10.1038/s41392-021-00475-w PMC7860167 33542177

[34] Wang C, Cao B, Liu Q, Zou Z, Liang Z, Gu L, et al. Oseltamivir compared with the Chinese traditional therapy maxingshigan-yinqiaosan in the treatment of H1N1 influenza: a randomized trial. Ann Intern Med 2011; 155: 217–25. doi: 10.7326/0003-4819-155-4-201108160-00005 21844547

[35] Hao P, Jiang F, Cheng J, Ma L, Zhang Y, Zhao Y. Traditional Chinese medicine for cardiovascular disease: evidence and potential mechanisms. J Am Coll Cardiol 2017; 69: 2952–66. doi: 10.1016/j.jacc.2017.04.041 28619197

[36] Li X, Zhang J, Huang J, Ma A, Yang J, Li W, et al. A multicenter, randomized, double-blind, parallel-group, placebo-controlled study of the effects of qili qiangxin capsules in patients with chronic heart failure. J Am Coll Cardiol 2013; 62: 1065–72. doi: 10.1016/j.jacc.2013.05.035 23747768

[37] Jiang K, Song Q, Wang L, Xie T, Wu X, Wang P, et al. Antitussive, expectorant and anti-inflammatory activities of different extracts from Exocarpium Citri grandis. J Ethnopharmacol 2014; 156: 97–101. doi: 10.1016/j.jep.2014.08.030 25178947

[38] Peng Y, Hu M, Lu Q, Tian Y, He W, Chen L, et al. Flavonoids derived from Exocarpium Citri Grandis inhibit LPS-induced inflammatory response via suppressing MAPK and NF-κB signalling pathways. Food Agr Immunol 2019; 30: 564–80. doi: 10.1080/09540105.2018.1550056

