# Supplementary material for: Discovery of pharmacological effects and targets of Citri Grandis Exocarpium based on SYSTCM and virtual screening
Source: Food Nutr Res. 2024 Jun 20;68:10.29219/fnr.v68.10618. doi: 10.29219/fnr.v68.10618 (PMC11227261; doi:10.29219/fnr.v68.10618)
Supplement: Supplementary file 1 [file FNR-68-10618-s1.docx]

### Table S1 The prediction heatmap of pharmacological effects of CGE

### Table S2 The heatmap of target profiles of compounds

### Table S3 Potential targets of CGE with fit value more than 0.9 of one compound

### Table S4 Literature search results of potential targets and anti-inflammatory and anti-allergic effects

### Table S5 Molecular docking of MAPK14 and MMP9

### Table S6 Molecular docking and pharmacophore results of compounds from CGE
